# Creating and curating a community of practice: introducing the evidence synthesis Hackathon and a special series in evidence synthesis technology

**DOI:** 10.1186/s13750-020-00212-w

**Published:** 2020-11-19

**Authors:** Neal R. Haddaway, Martin J. Westgate

**Affiliations:** 1 Mercator Research Institute On Global Commons and Climate Change, Torgauer Str. 19, 10829 Berlin, Germany; 2 Stockholm Environment Institute, Linnégatan 87D, Stockholm, Sweden; 3 Africa Centre for Evidence, University of Johannesburg, Johannesburg, South Africa; 4 Fenner School of Environment and Society, Australian National University, Acton ACT 2601, Australia

**Keywords:** Systematic review tools, Machine learning, Review technology, Efficiency, Digitisation

## Abstract

Evidence synthesis is a vital part of evidence-informed decision-making, but high growth in the volume of research evidence over recent decades has made efficient evidence synthesis increasingly challenging. As the appreciation and need for timely and rigorous evidence synthesis continue to grow, so too will the need for tools and frameworks to conduct reviews of expanding evidence bases in an efficient and time-sensitive manner. Efforts to future-proof evidence synthesis through the development of new evidence synthesis technology (ESTech) have so far been isolated across interested individuals or groups, with no concerted effort to collaborate or build communities of practice in technology production. We established the evidence synthesis Hackathon to stimulate collaboration and the production of Free and Open Source Software and frameworks to support evidence synthesis. Here, we introduce a special series of papers on ESTech, and invite the readers of environmental evidence to submit manuscripts introducing and validating novel tools and frameworks. We hope this collection will help to consolidate ESTech development efforts and we encourage readers to join the ESTech revolution. In order to future-proof evidence synthesis against the evidence avalanche, we must support community enthusiasm for ESTech, reduce redundancy in tool design, collaborate and share capacity in tool production, and reduce inequalities in software accessibility.

Evidence synthesis is a vital part of evidence-informed decision-making, and the substantial increase in the publication of systematic reviews and maps in recent years highlights that rigorous review is increasingly valued by the academic community. At the same time, however, the continuing explosion of evidence—a seemingly exponential growth in the volume of research evidence over the last few decades, referred to be some as an ‘infodemic’ [1]—will make efficient evidence synthesis increasingly challenging because of the necessary workloads (Fig. 1).

**Fig. 1:**
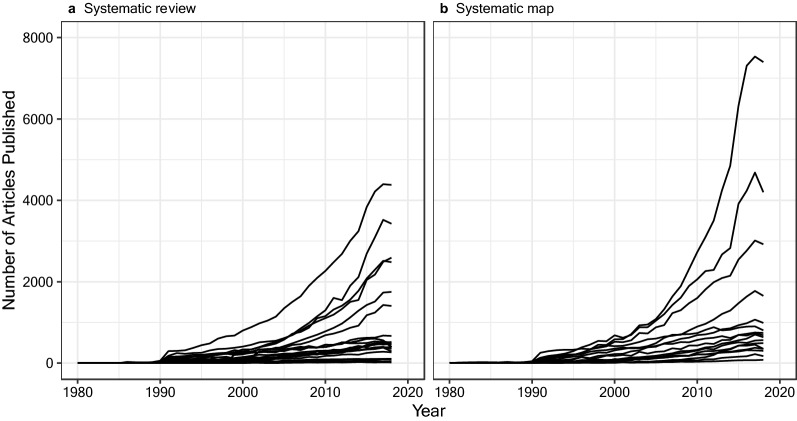
Number of search results per year for systematic reviews and maps. Each line represents a separate search conducted for this commentary and replicates an identical search from a systematic map or review published between 2012 and 2017 in the journal *Environmental Evidence*. Data and code are available on figshare; https://doi.org/10.6084/m9.figshare.12254321

As the appreciation and need for timely and rigorous evidence synthesis continue to grow, so too will the need for tools and frameworks that support users to conduct reviews of expanding evidence bases in an efficient and time-sensitive manner [2]. Such ‘evidence synthesis technology’ (ESTech) already exists in many forms, including: a range of systematic review management tools [3, 4]; machine learning algorithms for predicting relevance during screening [5]; and, tools to visualise evidence bases in heat maps and evidence atlases [6]. However, efforts to future-proof evidence synthesis through ESTech developments have so far been isolated across interested individuals or groups, with no concerted effort to collaborate or build communities of practice in technology production [7]. Developers typically produce ESTech solutions without consideration for: redundancy and overlap across similar tools; the need for continued support; the need for free and open access options; and a bias towards development of technology to suit Western consumers and those in the Global North (e.g. those with high speed internet).

In 2017, we established the evidence synthesis Hackathon (ESH; www.eshackathon.org) to act as a community of practice revolving around Open Science principles in evidence synthesis (Open Synthesis [8]). The mission of the ESH is to:

- Support the development, testing and promotion of new software and workflows;
- Build networks and capacity among researchers, practitioners and developers;
- Advocate for open synthesis.

Through our hackathons—highly interactive workshops for evidence synthesis experts and software programmers—we aim to produce workflows and tools that are open, reproducible, based on the best available technology and methods, and supported by the community. Primarily, we hope to continue to establish and support a community of practice working on ESTech to support collaborative working towards our goals and aims.

To date, we have had four hackathons (see https://www.eshackathon.org/events.html) in Sweden, Australia, and remotely, across a suite of evidence synthesis methods themes and specific stages (data visualisation), and including both programming and discussion streams. Some 20 projects have been initiated (see https://www.eshackathon.org/projects.html), and several tools are now publicly available and in use [6, 9]. Examples of some influential tools produced to date include: EviAtlas, a tool for producing interactive (geographically explicit) evidence atlases [6]; RobVis, a tool for producing risk of bias visualisations [9]; and metafor reporter, a function within the R package metafor [10] for automatically generating methods and results text from a meta-analysis model input (https://wviechtb.github.io/metafor/reference/reporter.html).

Based on our experiences across multiple ESH events, we recognise the following areas that are in particular need of technological development:

- Interoperability across different tools that would support users moving between ESTech options for different processes in their evidence syntheses;
- Improved efficiency and transparency in research discovery when searching for and exporting results from bibliographic databases and other sources of evidence;
- (Semi-)automated extraction of meta-data (descriptive information) and data from full texts in a reliable manner.

This is by no means an exhaustive list, but it highlights the range of challenges and solutions needed to move towards a more effective and fit-for-purpose ESTech and evidence synthesis landscape.

The ESH series has events planned for 2021 and beyond, with a primary emphasis on remote participation and inclusion of low- and middle- income country participants. In response to the COVID-19 pandemic, in 2020 we trialled fully online events for the first time.

The ESH will focus on:

- Finding a balance between integration of existing software and innovation through the production of novel tools;
- Building for a fit-for-purpose future evidence synthesis environment rather than retrofitting the present and past;
- Creating and curating an inclusive, collaborative and supportive community of practice of evidence synthesis technologists.

We introduce here an ongoing and open special series in environmental evidence, in association with the Collaboration for environmental evidence and the Campbell Collaboration. The series is a joint endeavour across environmental evidence and the Campbell Collaboration journal, Campbell systematic reviews. Authors should direct their presubmission enquiries to the dedicated ESTech special series website, which describes the series in full: https://estechseries.github.io/.

Readers are encouraged to submit commentaries (for example, that discuss barriers to the use of ESTech in resource-constrained contexts), methodologies (for example, introducing a novel tool and demonstrating its application in a real setting), and reviews (for example, a systematic review of review management tools). Authors should think carefully about the legacy of their work in the rapidly changing landscape of ESTech, and are encouraged to make use of online supplementary media to ensure their work remains up-to-date wherever possible; for example, providing a list of ESTech resources for a particular task that can be regularly updated.

The series aims to cover all stages of evidence synthesis processes; from planning, through conduct, to communication. We are also interested in issues relevant to ESTech that relate to other forms of evidence synthesis than systematic reviews and systematic maps (for example, rapid reviews and synopses), although relevance to rigorous evidence synthesis methods must be demonstrated.

The subject scope is not limited to environmental evidence synthesis and can span any subject where discipline agnostic ESTech can be discussed. We will publish papers on all aspects of ESTech including but not limited to: technology development; coordination and communities of practice; technology application in evidence syntheses; technology validation; acceptability and uptake of technology.

We hope this collection will help to consolidate ESTech development efforts and we encourage readers to join the ESTech revolution. We encourage papers that fulfil the following criteria:

- Technologies that fill a real gap: i.e., it should introduce a new tool that did not previously exist; or, make an existing tool much easier to use or make it available to a new audience.
- Technologies that are broadly accessible, as appropriate for the tool in question: i.e. they should pass standard checks to ensure they work across a range of operating systems or computational contexts; they must be free-to-use (or means-based, e.g. free for low- and middle- income country users) and preferably Open Source.

All submissions that meet these criteria will be considered, regardless of whether they include software or research from ESH events. Moreover, while many within the evidence synthesis community already share some or all of the goals that we have discussed, we call on the readers of environmental evidence to embrace ESTech and these ideals and goals in their future work.

In order to future-proof evidence synthesis against the evidence avalanche, we must support community enthusiasm for ESTech, reduce redundancy in tool design, collaborate and share capacity in tool production, and reduce inequalities in software accessibility.

## Data Availability

Data and code for Fig. 1 are available on FigShare; https://doi.org/10.6084/m9.figshare.12254321.
